# Supplementary material for: Speed-Selectivity in Retinal Ganglion Cells is Sharpened by Broad Spatial Frequency, Naturalistic Stimuli
Source: Sci Rep. 2019 Jan 24;9:456. doi: 10.1038/s41598-018-36861-8 (PMC6345785; doi:10.1038/s41598-018-36861-8)
Supplement: Supplementary file 1 — Supplementary Information [file 41598_2018_36861_MOESM1_ESM.pdf]

Speed-Selectivity in Retinal Ganglion Cells is Sharpened by Broad Spatial Frequency, Naturalistic Stimuli: Supplementary Material

César R Ravello[1*], **Laurent U Perrinet**[2], **María-José Escobar**[3*], **and Adrián G Palacios**[1*]

[1]Centro Interdisciplinario de Neurociencia de Valparaíso, Universidad de Valparaiso, Chile
[2]Institut de Neurosciences de la Timone, Aix Marseille Université / CNRS, France
[3]Universidad Técnica Federico Santa María, Departamento de Electrónica, Chile
[*]cesar.ravello@cinv.cl, mariajose.escobar@usm.cl, adrian.palacios@uv.cl

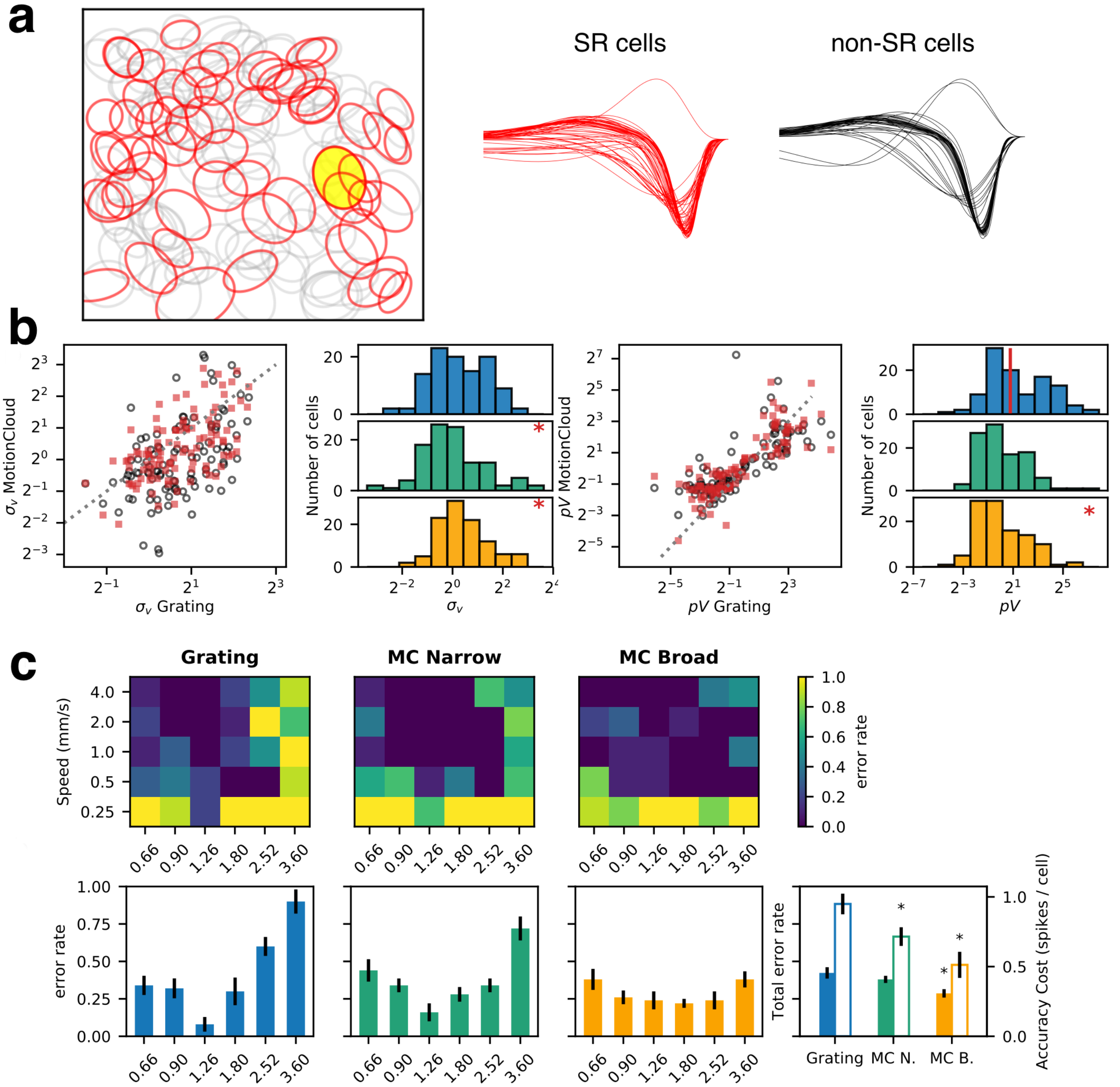

Figure S1. Main results from a second retina. **a** Receptive Field characterization of SR and non-SR cells, as in **Fig. 4** of the main text. **b** Changes in tuning properties when stimulated with MC. The scatter plot shows that a large proportion of points falls below the equality line, meaning that the width of the tuning curve (σ_v) is larger when stimulated with gratings ($p < 0.005$, Wilcoxon signed-rank test), while the distributions of preferred speed does not change ($p < 0.05$ only for the MC broad stimuli), consistent with the results shown in **Fig. 5** of the main text. **c** Decoding performance increases for the MC stimuli. The 2-D plots show the error rate at each condition for each type of stimulus, while the bar plots show the aggregated error rates for each spatial frequency. In the rightmost plot, solid columns show total error rate and open bars show the Accuracy cost, that is significantly lower for the MC stimuli ($p < 0.01$, Wilcoxon signed-rank test)
